# C3 deficiency ameliorates the negative effects of irradiation of the young brain on hippocampal development and learning

**DOI:** 10.18632/oncotarget.8400

**Published:** 2016-03-28

**Authors:** Marie Kalm, Ulf Andreasson, Thomas Björk-Eriksson, Henrik Zetterberg, Milos Pekny, Kaj Blennow, Marcela Pekna, Klas Blomgren

**Affiliations:** ^1^ Center for Brain Repair and Rehabilitation, Institute of Neuroscience and Physiology, University of Gothenburg, Gothenburg, Sweden; ^2^ Clinical Neurochemistry Laboratory, Institute of Neuroscience and Physiology, University of Gothenburg, Sahlgrenska University Hospital, Mölndal, Sweden; ^3^ Department of Oncology, Sahlgrenska University Hospital, Gothenburg, Sweden; ^4^ Department of Molecular Neuroscience, UCL Institute of Neurology, Queen Square, London, UK; ^5^ Florey Institute of Neuroscience and Mental Health, Parkville, Victoria, Australia; ^6^ Hunter Medical Research Institute, University of Newcastle, New South Wales, Australia; ^7^ Department of Women's and Children's Health, Karolinska Institute, Karolinska University Hospital, Stockholm, Sweden

**Keywords:** cranial radiotherapy, complement system, neurogenesis, neuroinflammation, late effects

## Abstract

Radiotherapy in the treatment of pediatric brain tumors is often associated with debilitating late-appearing adverse effects, such as intellectual impairment. Areas in the brain harboring stem cells are particularly sensitive to irradiation (IR) and loss of these cells may contribute to cognitive deficits. It has been demonstrated that IR-induced inflammation negatively affects neural progenitor differentiation. In this study, we used mice lacking the third complement component (*C3*^−/−^) to investigate the role of complement in a mouse model of IR-induced injury to the granule cell layer (GCL) of the hippocampus. *C3*^−/−^ and wild type (WT) mice received a single, moderate dose of 8 Gy to the brain on postnatal day 10. The *C3*^−/−^ mice displayed 55 % more microglia (Iba-1+) and a trend towards increase in proliferating cells in the GCL compared to WT mice 7 days after IR. Importantly, months after IR *C3*^−/−^ mice made fewer errors than WT mice in a reversal learning test indicating better learning capacity in *C3*^−/−^ mice after IR. Notably, months after IR *C3*^−/−^ and WT mice had similar GCL volumes, survival of newborn cells (BrdU), microglia (Iba-1) and astrocyte (S100β) numbers in the GCL. In summary, our data show that the complement system contributes to IR-induced loss of proliferating cells and maladaptive inflammatory responses in the acute phase after IR, leading to impaired learning capacity in adulthood. Targeting the complement system is hence promising for future strategies to reduce the long-term adverse consequences of IR in the young brain.

## INTRODUCTION

Radiotherapy (RT), an effective tool in the treatment of malignant tumors, is used not only in adult patients but also in children suffering from primary or metastatic brain tumors and central nervous system (CNS) involvement of leukemia or lymphoma. In addition, whole body irradiation, including the brain, is part of some protocols prior to hematopoietic stem cell transplantation. Damage to normal, surrounding brain tissue constitutes a major problem and RT is associated with adverse side effects, particularly in pediatric patients [1]. Intellectual impairment as well as perturbed growth and puberty are some of the side effects seen after RT [2-6].

Ionizing radiation can produce free radicals and DNA damage, causing proliferative cells to undergo apoptosis or mitotic catastrophe. Mature neurons are considered to be in a permanent state of growth arrest, whereas stem and progenitor cells have a prominent proliferative capacity and are therefore highly vulnerable to irradiation (IR). Neurogenic niches, the subventricular zone (SVZ) and the subgranular zone (SGZ) of the dentate gyrus (DG) in the hippocampus, are highly susceptible to IR-induced injury. This has been demonstrated in rodents [7-10] and appears to be true also for humans [11]. In addition to acute cell damage and loss, IR can affect the survival of stem and progenitor cells, leading to a limited potential for repopulation or regeneration [7]. Earlier, we found that a sigle dose of 6-8 Gy to young rat [12] or mouse [13] brains (which corresponds well to the therapeutic doses used in the treatment of pediatric CNS tumors), produced a long-lasting decrease in hippocampal neurogenesis, more pronounced in rats than in mice [8]. A single dose of 8 Gy to the young rat [10] or mouse brain virtually abolished growth of the DG with no morphological recovery. In contrast, a study using a single dose of 4 Gy to adult mice found a reversible effect on proliferation and neurogenesis in the DG [14]. However, although radiation virtually abolished neurogenesis in both neurogenic niches in the acute phase, the long-term effects were profoundly different such that the IR effects on the DG were long-lasting, whereas SVZ neurogenesis appeared to recover with time [12]. These findings point to substantial differences between the immature and adult brain in the response to IR, something that we have shown already [15], as well as differences in the ability of the neurogenic regions to recover from the IR-induced damage. It has been shown that IR causes a change in the microenvironment, making the progenitor cells in the DG shift from neurogenesis to gliogenesis, and this was attributed to the inflammatory response [16].

**Figure 1:**
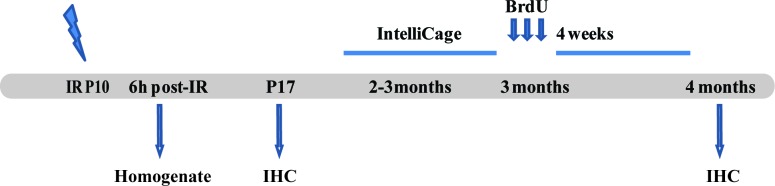
Outline of the experimental design The animals were irradiated on P10. The first group of animals was sacrificed 6 h post-IR to measure cytokine levels in the brain. The second group was sacrificed 7 days after IR and used for IHC. The third group of animals was placed in the IntelliCage when 2-3 months old. After the behavior testing they were given 3 injections of BrdU, then sacrificed 4 weeks later and used for immunohistochemistry. IHC = Immunohistochemistry.

The complement system is a potent mediator of inflammation, but it also has a range of non-immune functions in the CNS, including tagging synapses for elimination [17]. Within the CNS, the complement proteins are produced by neurons, oligodendrocytes, microglia and astrocytes [18]. C3 has a central position in the complement system and genetic ablation of C3 prevents activation of the complement system by any of the three activation pathways [19]. As we previously reported, transcription of the *C3* gene was highly up-regulated 6 hours after IR [20], and in this study we used *C3*^−/−^ mice to investigate the role of the complement system in the short- and long-term effects of IR (6 h to 4 months) on the developing brain. We assessed the morphology and inflammatory response in the GCL of the hippocampus, a brain region important for learning and memory, as well as the place learning, reversal learning and cell survival in the hippocampus (the timeline of the study is presented in Figure 1).

## RESULTS

### Effects of C3 deficiency in naïve mice

The protein concentration of keratinocyte chemoattractant (KC) in brain homogenates was significantly lower in *C3*^−/−^ compared to WT mice at the age of P10 (Table 1). At P17 the GCL volume as well as the numbers of proliferating cells (PPH3+), immature neurons (NeuroD+), microglia (Iba-1+) and astrocytes (S100β+) did not differ between *C3*^−/−^ and WT mice. There were no differences between the WT and *C3*^−/−^ mice at the age of 4 months in the total number of surviving cells (BrdU-labeled), immature neurons (Dcx+), microglia (Iba-1+), or astrocytes (S100β+, Table 1). The effects of C3 deficiency on neurophysiology and behavior in adult mice have been described previously [21]. In brief, constitutive absence of C3 is associated with an increased number of functional excitatory synapses in the hippocampal CA1 region and enhanced place and reversal learning in adult mice. Recently, aged *C3*^−/−^ mice were reported to have enhanced cognition and LTP as well as reduced anxiety [22].

**Table 1:** effects of C3 deficiency in naïve mice

			WT			*C3*			
			Mean	SEM	*N*	Mean	SEM	*N*	*P*
**P 10**	**Cytokines**	mKC (pg/mg)	1.5	0.06	6	1.3	0.05	5	0.03
		mCCL2 (pg/mg)	1.5	0.09	6	1.3	0.06	5	n.s
		mVEGF (pg/mg)	3.8	0.05	6	3.6	0.06	5	n.s
		mIL-1b (pg/mg)	0	0	6	0.1	0.07	5	n.s
		mIL-6 (pg/mg)	0.2	0.1	6	0	0	5	n.s
**P 17**	**Volume**	GCL (mm^3^)	0.19	0.008	6	0.21	0.02	7	n.s
	**PHH3**	Total cells	1,298	112	6	1,321	236	6	n.s
	**Neuro D**	Total cells	12,317	1,892	6	16,195	2,479	6	n.s
	**Iba-1**	Total cells	2,270	263	6	2,699	334	6	n.s
	**S100β**	Total cells	2,976	243	6	3,598	473	6	n.s
**4 Months**	**Volume**	GCL (mm^3^)	0.26	0.006	8	0.23	0.003	8	n.s
	**BrdU**	Total cells	323	28	8	381	52	7	n.s
	**DCX**	Total cells	3,313	137	8	3,687	364	7	n.s
	**Iba-1**	Total cells	2,484	186	7	3,111	229	7	n.s
	**S100β**	Total cells	4,491	128	8	4,236	330	7	n.s

### Effects of C3 deficiency following IR

#### The acute phase: inflammatory markers

**Figure 2:**
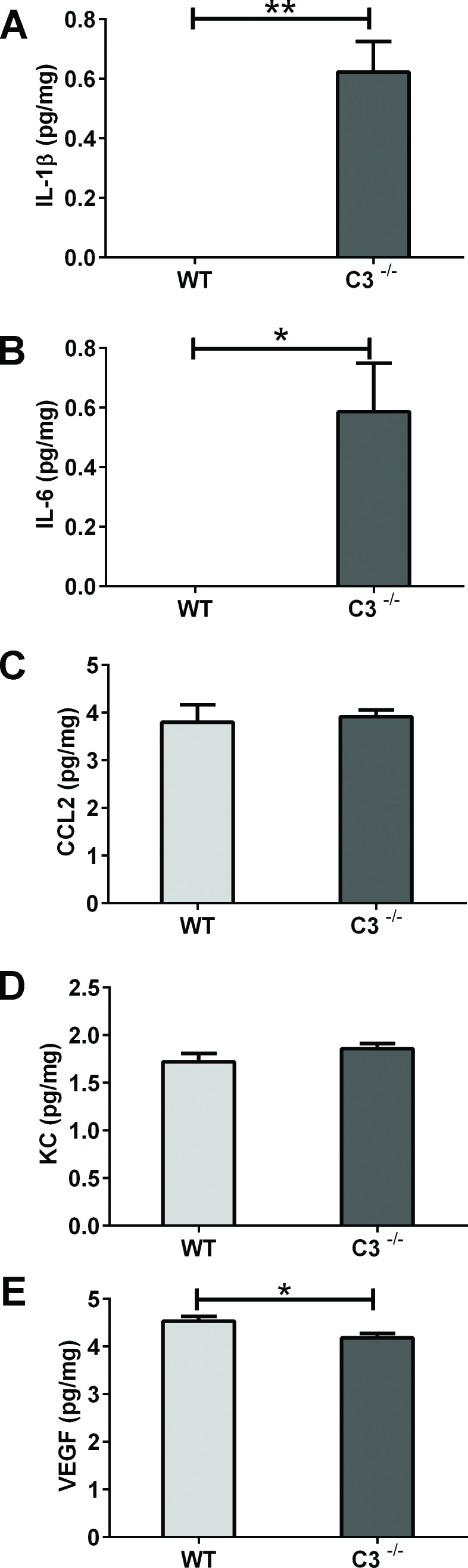
The acute phase after IR Six hours after IR, brains were collected, homogenized and analyzed using multiplex immunoassay. Concentrations of IL-1β **A.** IL-6 **B.**, CCL2 **C.**, KC **D.** and VEGF **E.** are presented as mean concentration (pg/mg) protein ± SEM and *n* = 5-7. * Mann-Whitney U-test was performed to test differences between genotypes. *P* < 0.05*, *P* < 0.01**, WT = wild type mice, *C3*^−/−^ = C3 deficient mice, SEM = standard error of the mean.

The protein concentrations of interleukin (IL)-1β, IL-6, chemokine (C-C motif) ligand 2 (CCL2), KC, and vascular endothelial growth factor (VEGF) were measurable in brain homogenates 6 hours after IR. After IR, the concentration of IL-1β was higher in *C3*^−/−^ than in WT brains (Figure 2A, *P* < 0.01), with IL-1β being below the detection limit in WT brains. Similar results were obtained for IL-6 (Figure 2B, *P* < 0.05). There were no differences between *C3*^−/−^ and WT mice in the expression of CCL2 or KC (Figure 2C-2D). The expression of the growth factor VEGF was marginally lower in *C3*^−/−^ mice compared to WT mice (Figure 2E, *P*< 0.05).

#### The subacute phase: IR-induced morphological changes

**Figure 3:**
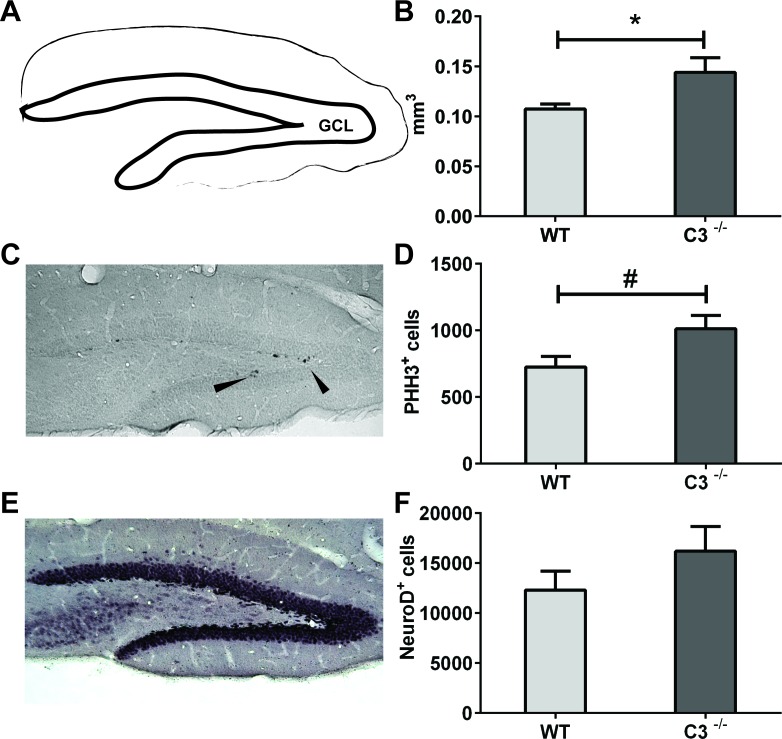
IR-induced changes during the subacute phase The volume of GCL **A.** was measured at P 17 (7 days after IR). **B.** the average volumes of the GCL. **C.** A representative microphotograph of phospho-histone H3-positive (PHH3^+^, proliferating) cells from an irradiated wild type GCL. Arrowheads show immunopositive cells. **D.** The total numbers of PHH3^+^ cells in the GCL after IR. **E.** A representative microphotograph of Neuro D, (neural progenitors and immature neurons) cells from an irradiated wild type GCL. **F.** The total numbers of Neuro D^+^ cells in the GCL after IR. Data are shown as mean ± SEM, *P < 0.05, #*P* = 0.05, *n* = 5-6 per group. WT = wild type mice, C3^−/−^ = C3-deficient mice, SEM = standard error of the mean.

IR-induced ablation of neurogenesis in the young hippocampus results in decreased, or even abolished, growth of the GCL [8, 10, 13, 23-26]. We therefore assessed the volume of the GCL 7 days after IR. We found that after IR the *C3*^−/−^ mice had a 34 % larger GCL compared to WT mice, indicating that the *C3*^−/−^ mice are protected to some extent from the IR-induced growth impairment (Figure 3A-3B, *P* < 0.05).

The *C3*^−/−^ mice showed a trend towards higher numbers of phospho-histone H3-positive (PHH3^+^) cells, i.e. cells proliferating at the time of sacrifice, in the GCL after IR (Figure 3C-3D, *P* = 0.0500). The numbers of Neuro D-positive cells in the GCL, reflecting the number of neural progenitors and immature neurons (Figure 3E-3F), were not different between irradiated WT and *C3*^−/−^ mice.

**Figure 4:**
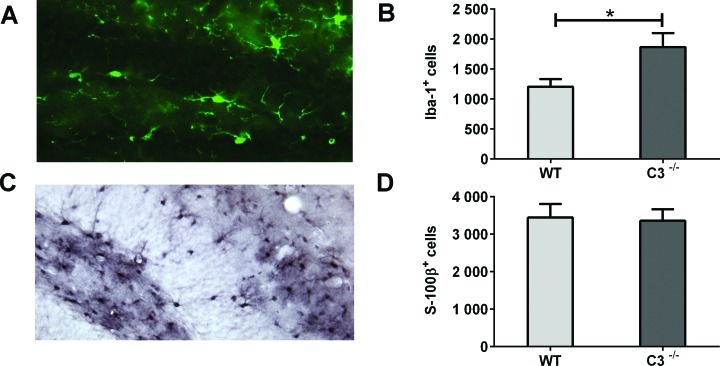
IR-induced changes in the microenvironment during the subacute phase **A.** A representative microphotograph of Iba-1-positive cells (microglia) in the GCL. **B.** The average numbers of Iba-1-positive cells in the GCL. **C.** A representative microphotograph of S100β-positive cells (astrocytes) in the DG. **D.** The average numbers of S100β-positive cells in the GCL. Data are shown as mean ± SEM *P < 0.05, *n* = 6 per group. WT = wild type mice, C3^−/−^ = C3-deficient mice, SEM = standard error of the mean.

The pro-inflammatory microenvironment post-IR is known to decrease neurogenesis and increase the number of glial cells [16]. We quantified microglia and astrocytes 7 days after IR (Figure 4) and found that *C3*^−/−^ mice had 55 % more microglia in the GCL compared to WT mice (Figure 4B, *P* < 0.05). The numbers of astrocytes did not differ between the two groups of mice (Figure 4C-4D). The increased number of proliferating cells could not be explained by an increase in microglia, however, because less than 0.2 % of the PPH3^+^ cells were also positive for Iba-1, with no difference between WT and *C3*^−/−^ mice.

### Learning

**Figure 5:**
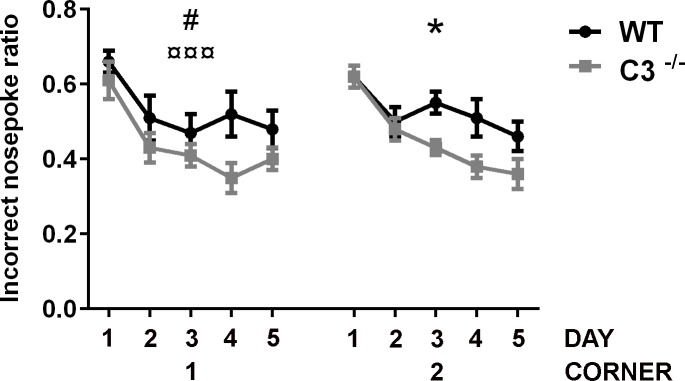
Functional effects of IR in the absence of C3 The effects on memory and learning were investigated using the IntelliCage platform. The figure shows the incorrect nose poke ratio. The x-axis shows the number of days (1-5) for the first and second allocated corners. The first corner reflects place learning and the second corner reflects reversal learning. GEE analysis was used for statistical analysis. Data are shown as mean ± SEM. WT = wild type irradiated (*n* = 14), C3^−/−^ = C3-deficient irradiated (*n* = 11), SEM = standard error of the mean. *p* < 0.05^*,#^, *p* < 0.001^¤¤¤^ (# = genotype, ¤ = time, * = interaction).

To investigate the possible functional effects of IR in the absence of C3, the IntelliCage platform was used 2-3 months after IR. The incorrect nose poke ratio (the ratio of attempts to open the door in non-allocated, incorrect corners, to attempts to open the allocated, correct corner) was used as a measure of the animal's ability to learn that it cannot open the door in such a corner, i.e. there was an inverse relation between memory retention and the incorrect nose poke ratio in non-allocated to allocated corners. Both groups of mice showed that they understood the task and improved over time (*P* < 0.05, Figure 5). Both groups displayed a positive learning curve during the first corner period, but the *C3*^−/−^ mice had a 40 % better improvement in odds ratio per day compared to the WT mice (*P* < 0.05) indicating better place learning. During the second corner learning period, where reversal learning takes place, there was a difference in the incorrect nose poke ratios over time between the groups (*P* < 0.05); Whereas the WT mice improved 11.5 % per day, the *C3*^−/−^ mice improved 21.4 % per day. Together, these results indicate better learning capacity in *C3*^−/−^ mice after IR.

### Long-term effects on a cellular level

At the age of 4 months, the long-term effects of IR were evaluated in the GCL. The difference in GCL volume observed between WT and *C3*^−/−^ mice 7 days after IR was no longer detected (0.164 ± 0.002mm^3^ and 0.166 ± 0.005mm^3^, respectively). The two groups of mice did not differ in cell survival, measured as total number of BrdU-positive cells in the GCL (45 ± 13.6 BrdU^+^-cells in the WT mice and 65 ± 8.2 BrdU^+^-cells in the *C3*^−/−^ mice (n.s.)). Due to the low numbers of BrdU-positive cells at this late time point, neurogenesis could not be assessed by counting the BrdU^+^/NeuN^+^ cells, but the total numbers of Dcx-positive cells did not differ between the *C3*^−/−^ (229.5±110 Dcx^+^-cells) and WT mice (241.7 ± 119 Dcx^+^-cells). Further, the numbers of microglia and astrocytes were comparable between the two groups of mice. The numbers of Iba-1^+^ cells were 1,666 ± 168 in the WT and 1,914 ± 184 in *C3*^−/−^ mice (n.s.). The numbers of astrocytes (S-100β+-cells) were 3,402 ± 165 in WT and 3,613 ± 231 in *C3*^−/−^ mice (n.s).

## DISCUSSION

In this study, we investigated the role of C3 in IR-induced impairment of hippocampal growth and learning in young mice. We assessed the effects of C3 deficiency 6 hours (acute phase), 7 days (subacute phase) and several months after cranial IR. Our major findings were that C3 deficiency was associated with (i) higher levels of pro-inflammatory cytokines during the acute phase, (ii) higher numbers of microglia and a trend towards higher cell proliferation in the GCL during the subacute phase, and (iii) better learning capacity after IR.

During the acute (initial 2-12 hours) response to IR, acute cell death in the neurogenic region coincides with a transient production of pro-inflammatory chemokines and cytokines in the young rat brain [8, 20]. Similarly, CCL2 was up-regulated both in WT and *C3*^−/−^ mice after IR. In contrast, IL-1β and IL-6 were up-regulated only in C3-deficient brains. This appears paradoxical, in light of the detrimental effects on neurogenesis attributed to IL-6 after IR in the adult rat hippocampus [27-29]. The immature brain, however, reacts differently, as evidenced by the lack of IL-6 observed after IR in the WT mouse brain in this study and unchanged or decreased levels in the immature rat brain [15, 20]. Indeed, in a recent study, we showed that IL-6 decreased after IR to the juvenile but not the adult rat brain [15], supporting the notion that the young and adult brain respond differently to IR. As IL-6 levels increase during normal brain growth [15], IL-6 could play a role in brain development. There is also experimental evidence that IL-6 after injury could have beneficial, neuroprotective effects in the brain [30, 31] as well as promote axon regeneration and functional recovery [32]. While IL-6 was reported to promote glial differentiation of neural precursor cells [27, 28], it can also promote neuronal differentiation [33, 34]. It should also be noted that IL-6 plays an important role in cell maturation outside the brain by affecting B- and T-cells, hepatocytes, and hematopoietic progenitor cells [35]. It remains to be further investigated if the IL-6 level changes are governed by cells in the CNS or in the periphery, and whether they reflect pro- or anti-inflammatory reactions in the CNS. IL-1β is an early, upstream cytokine in the signaling cascades of neuroinflammation. As IL-1β can be detected as early as 15 minutes after cortical injury [36], it is possible that 6 h after IR was too late to detect IL-1β expression in the WT brains in the present study. The increase in IL-1β levels observed in the *C3*^−/−^ brains can, similar to IL-6, represent a reaction opposite to that observed in WT brains or can be caused by a delayed or stronger expression, thereby remaining detectable 6 h post-IR. As IL-1β can promote the expression of IL-6 [37], it is possible that these two pro-inflammatory are expressed in sequence. The major source of IL-1β in the injured brain is microglia [38], which appear to respond differently to IR in the adult and the immature brain [12, 15, 28, 39]. Our present findings show that higher levels of IL-1β and IL-6 early after IR are associated with better outcome, although the underlying mechanisms remain to be elucidated.

The DG, in particular the proliferating cells in the SGZ of the GCL, are known to be affected by IR. This may occur in at least three ways: through cell death (apoptosis or mitotic catastrophe), through decreased proliferation and through perturbed precursor cell differentiation [8, 10, 13, 16, 26, 40]. It has been shown that the volume of the GCL is smaller 7 days after IR in young rats and mice at ages when the hippocampus is still growing [8]. Given the limited amount of cell death, this difference is most likely due to decreased or even abolished growth of the GCL [10], which in turn can be explained, at least partly, by decreased or abolished neurogenesis [10, 13, 26]. Furthermore, it has been demonstrated that the fate of surviving hippocampal precursor cells changes after IR of the adult rat brain, favoring a glial phenotype [16], thereby contributing to the lower final number of neurons in this region. In addition to the acute death of proliferating cells after IR, it has been shown that IR can influence the proliferative potential of the surviving precursor cells, leading to a limited capacity for repopulation or regeneration [7, 13]. The lack of growth is therefore caused by a combination of a loss of cells capable of generating new tissue and negative effects on cells that survived the insult. Here we show that in the subacute phase (7 days) after IR, *C3*^−/−^ mice had larger GCLs accompanied by higher levels of cell proliferation. Thus, the tissue response in the GCL appears to be less detrimental in mice devoid of C3. Our finding of higher numbers of microglia in the GCL of irradiated *C3*^−/−^ mice suggests that microglia ameliorate the toxic response of IR and merits further investigation of microglial functions in the IR-affected regions. In our earlier studies we have shown that a limited number of microglia die after IR [39] and that microglia react differently to irradiation in the juvenile and the adult brain [15], likely affecting both the response to IR and brain development. Since the *C3*^−/−^ mice have both higher proliferation and a better learning outcome, our data suggest that a higher number of microglia is beneficial following IR to the young brain. Further, anti-inflammatory strategies can ameliorate some of the negative effects of IR [28, 41, 42] and our results point to the complement system as an important mediator of the IR-induced inflammation and hippocampal growth impairment.

We observed that the irradiated *C3*^−/−^ mice performed better than controls in both learning and reversal learning, a test that can be regarded as, at least partly, hippocampal-dependent [43], about 3 months after IR. Our results demonstrate a difference in the ability to replace one declarative memory (learning) with a new, modified one (reversal learning). This is analogous to the Morris Water Maze, which entails both a procedural part (swimming to find the platform) and a declarative part (finding the correct quadrant) and where reversal learning involves changing the declarative memory to a new one (when the position of the platform is changed) while maintaining the procedural memory [44]. In the IntelliCage, both WT and *C3*^−/−^ mice continuously learn to perform correct visits and improve over time, both in the learning and reversal learning phases (Figure 5), indicating no impairment of the procedural memory. Declarative memory, including spatial memory, is linked to the hippocampus [45], including the creation of a new declarative memory during reversal learning, but it is likely that other regions of the brain (e.g., the prefrontal cortex, the entorhinal cortex, amygdala, and striatum) also take part in this specific type of learning task. IR of the juvenile rodent brain is known to affect cognitive performance and other types of behavior [13, 20, 23, 25, 46-48]. Neurogenesis has been demonstrated to be important for hippocampal-dependent learning [49, 50]. It has also been shown that IR-induced changes in neurogenesis were associated with spatial memory retention deficits determined using the Morris water maze [51, 52]. In the late phase (months after IR), the GCL volumes, the proliferative capacity as well as the numbers of immature neurons, microglia and astrocytes did not differ between WT and *C3*^−/−^ mice. These results suggest that the cognitive performance of mice, as assessed by the IntelliCage system, is determined also by other brain regions such as corpus callosum that is strongly affected by IR [10, 53]. Corpus callosum, the largest white matter commissure, is affected in children subjected to cranial RT, with the greatest effects observed in the most posterior subregions (isthmus and splenium) [54]. It remains to be determined if the white matter and/or other regions are involved in the better learning capacity displayed by *C3*^−/−^ mice after IR. Cognition is dependent on aspects of brain plasticity other than cell genesis, such as the density and function of neuronal synapses. Naïve adult *C3*^−/−^ mice show increased number of functional excitatory synapses in the hippocampal CA1 region and enhanced place and reversal learning [21] despite reduced basal neurogenesis in the DG as well as the SVZ [55]. The *C3*^−/−^ mice in this study were therefore at an advantage already before the IR-induced injury, and interestingly they continued perform virtually as well as non-irradiated C3 mice. Thus the mechanisms by which C3-mediated mechanisms modulate hippocampal function and tissue response to IR may go well beyond cell proliferation and neurogenesis.

In summary, we have shown that growing hippocampi devoid of C3 show less adverse effects of IR, including better cognitive performance in adulthood. C3 is an important mediator of IR-induced impairment of early hippocampal growth and function.

## MATERIALS AND METHODS

### Animals

*C3*^−/−^ mice were produced as described earlier [19] and backcrossed on a C57BL/6 background (Charles River Laboratories, Sulzfeld, Germany) for 13 generations. Wild type (WT) C57BL/6 mice served as controls (Charles River Laboratories, Sulzfeld, Germany). The animals were kept on a 12-hour light cycle where food and water was provided *ad libitum.* The mice for the acute and subacute phases were kept with their dams until sacrifice. The mice for the later time point were after weaning, kept in groups of up to 10, separated by genotype and gender. Before weaning, all mice were sedated with isoflurane and implanted subcutaneously with microtransponders (DATAMARS, PetLink, Youngstown, USA) to be able to identify individual animals in the IntelliCages. All mice that were in the IntelliCages were given daily injections of bromodeoxyuridine (BrdU; 50 mg/kg) for three consecutive days four weeks before the animals were euthanized. All animal experiments were approved by the local committee of the Swedish Animal Welfare Agency (30/2008)

### Irradiation procedure

For IR a linear accelerator (Varian Clinac 600 CD; Radiation Oncology Systems LLC, San Diego, CA, USA) with 4 MV nominal photon energy and a dose rate of 2.3 Gy/min was used. Postnatal day 10 (P10) pups of both sexes (equal distribution of males and females between groups) were anaesthetized with an i.p. injection of 50 mg/kg tribromoethanol (Sigma, Stockholm, Sweden). The animals were placed in prone position (head to gantry) on an expanded polystyrene bed. The whole brain was irradiated with a radiation field of 2 × 2 cm. The IR source to skin distance was ~99.5 cm. The head was covered with a 1 cm tissue equivalent material to obtain an even IR dose throughout the underlying tissue. The dose variation within the target volume was estimated to be ±5 %. The entire procedure was completed within 10 min. After IR, the pups were returned to their biological dams until sacrificed. Naïve mice were sham-irradiated and anaesthetized but not subjected to IR. A single absorbed dose of 8 Gy was administered. This dose is equivalent to approximately 18 Gy when delivered in repeated 2 Gy fractions, according to the linear-quadratic formula [56] and an α/β-ratio of 3 for late effects in the normal brain tissue. This represents a clinically relevant dose, equivalent to the total dose used in treatment protocols for prophylactic cranial IR in selected cases of childhood acute lymphoblastic leukemia. The doses used for malignant pediatric brain tumors are often higher, 35 Gy craniospinal IR with 55 Gy to the tumor bed.

### Immunohistochemistry

Animals were deeply anaesthetized with sodium pentobarbital (60 mg/ml, 2 ml/100 g body weight i.p.) and transcardially perfusion-fixed with Histofix (EMD Chemicals Inc., an Affiliate of Merck KGaA, Darmstadt, Germany) in 0.1 M phosphate-buffered saline (PBS). The brains were removed and immersion-fixed in the same solution at 4°C for 24 h. The left and right hemispheres were separated and put in 0.1 M phosphate-buffer with 30% sucrose, pH 7, for storage. The right hemisphere was cut in 25 μm sagittal sections on a sliding microtome. The short term brains (7 days after IR) were cut in a 10-series and the long term brains (4 months after IR) were cut in a 12-series. The sections were collected and stored in a cryoprotection solution containing 25% glycerine and 25% ethylene glycol. The sections were rinsed with tris-buffered saline (TBS) before incubation with 0.6% H_2_O_2_ in TBS for 30 minutes (only for sections treated with 3.3-diaminobenzidine at the end). Non-specific binding was blocked with 3% donkey serum (Jackson ImmunoResearch Laboratories Inc., Cambridgeshire, UK) in 0.1% Triton X-100 and TBS. Primary antibodies, rabbit anti-phospho-histone H3 (1:100, Upstate, Temecula, CA), rabbit anti-S100β (1:5,000, z 0311, Dako, Glostrup, Denmark), rat anti-BrdU (1:500, AbD Serotec, Kidlington, UK), goat anti-Dcx (1:125, Santa Cruz Biotechnology, INC, California, USA), rabbit anti-Iba-1 (1:1,000, 019-19741, WAKO Pure Chemical Industries, ltd., Osaka, Japan), and donkey anti-Iba-1 (confocal analysis, 1:500, Abcam, Cambridge, UK) were incubated in 3% donkey serum in 0.1% Triton X-100 and TBS over night at 4°C (sections stained for Dcx for 3 nights) and subsequently incubated with biotinylated donkey anti-rabbit or anti-goat secondary antibody (1:1,000 Jackson ImmunoResearch, West Grove, PA) in 3% donkey serum/ 0.1% TritonX-100/ TBS for 1 hr. Visualization was performed using an avidin-biotin-peroxidase solution (Vectastain ABC Elite kit, Vector Laboratories, Burlingame, CA). Stainings were developed with 3.3-diaminobenzidine. Donkey anti-rabbit (confocal), rabbit anti-PHH3 and Rabbit anti-Iba-1 was followed by incubation with a donkey anti-rabbit IgG 488- or 555-conjugated secondary antibody (1:1,000, Molecular Probes, Inc., Eugene OR, USA) for one hour and mounted with Prolong mounting medium (Molecular Probes, Inc., Eugene OR, USA) containing DAPI.

### Cell quantification

Cells were counted in serially cut sections in the granule cell layer (GCL) of the hippocampus using stereological principles (Stereoinvestigator, MicroBrightField, Colchester, VT, USA). The counting started when GCL had divided into a dorsal and a ventral part (in sagittal sections). Only the dorsal GCL was measured. All sections were counted from that starting point until the dorsal blade of the GCL had disappeared. For the NeuroD quantification the fractionator function was used in the Stereoinvestigator program. Every 10^th^ section was counted throughout the whole GCL 7 days after IR. Every 12^th^ section was counted throughout the whole GCL four months after IR. To get the total volume the sum of the area traced was multiplied by the section thickness and series number. The total number of cells was received by multiplying the counted cells with the series.

For analysis of newly generated microglia 50 PPH3^+^cells per animal was analyzed for coexpression of Iba-1 using a confocal laser scanning microscope (TCS SP2; Leica, Wetzlar, Germany).

### Immunoassay

IR and control animals were sacrificed by decapitation 6 h after IR (P10). The brains were quickly dissected out, frozen in isopentane and dry ice and stored at −80°C. The entire brain was used to get enough material to analyze. We found in an earlier study that the cytokine concentrations were very similar in different brain regions after irradiation, thereby justifying the use of the entire brain to reflect the situation in the hippocampus [20]. The tissue was homogenized by sonication in 5 volumes of PBS containing 0.1 % Triton X-100, 0.5 % protease inhibitor cocktail (Sigma, Stockholm, Sweden) and 5 mM EDTA. Crude cytosolic fractions were produced by centrifuging homogenates at 20,000 × *g* for 10 min at 4°C, subsequently stored at −80°C. Total protein concentration was measured using a NanoDrop Spectrophotometer (Thermo Scientific, Wilmington, DE, USA). Crude cytosolic fractions were assayed for the following mouse proteins: keratinocyte chemoattractant (KC), chemokine (C-C motif) ligand 2 (CCL2), vascular endothelial growth factor (VEGF), interleukin (IL)-1 β, IL-6, granulocyte-macrophage colony-stimulating factor (GM-CSF) and tumor necrosis factor-α (TNF-α) using a custom-designed 7-plex assay (Meso Scale Discovery, Gaithersburg, Maryland). The cytokines and chemokines were selected based on our earlier studies. Only the measurable proteins are presented in the results section. GM-CSF and TNF-α were below the detection limits. Prior to analysis, the samples were centrifuged at 15,000 × *g* for 10 minutes to remove possible debris. The assay was performed according to the protocol from the manufacturer using a Sector^®^ Imager 6000 instrument (Meso Scale Discovery, Gaithersburg, Maryland) and the Discovery Workbench^®^ software (Meso Scale Discovery, Gaithersburg, Maryland) for reading and quantification, respectively.

### Testing of place and reversal learning using IntelliCage®

The IntelliCage platform (New Behavior, Zurich, Switzerland) for unbiased monitoring of mouse behavior in a home cage setting has been described elsewhere [48, 57-59]. The animals were housed in the IntelliCages in groups of up to 10 animals per cage. Females were 3 months and males 2 months old at the start of the IntelliCage experiment. The mice were from the same litters and males and females were tested separately and consecutively in the IntelliCages, hence the different ages. The experiment started with 5 days of introduction in the IntelliCages where the animals were acclimatized to drinking and performing nose pokes to gain access to the water bottles. The introduction period was followed by a corner learning period where each animal was randomized to one corner (the most visited corner during the introduction was excluded). This allocated corner was programmed as the correct corner while the other three corners were programmed as incorrect. The mice were only allowed to drink from the water bottles in the correct corner, where a nose poke would open a door and give them access to the two water bottles. In incorrect corners, the doors to the water bottles remained closed after nose pokes. After 5 days, the animals were randomized to a new corner (the previous corner excluded). The incorrect nose poke ration was calculated by dividing the number of nose pokes in non-allocated (incorrect) corners with the total number of nose pokes.

Animals that failed to drink in the IntelliCages or were not registered as visiting any corners at all (indicating misplaced or missing microtransponders) were removed from the IntelliCages. Special consideration was taken into differences between gender through having the same percentage of males and females in all the groups when analyzed. Food was provided *ad libitum* during the experiments in the IntelliCage and red plastic houses were provided as shelters. Data from the IntelliCages were analyzed using the IntelliCage software (IntelliCage Plus, 2.4, NewBehavior AG, Zurich, Switzerland) followed by statistical analysis. Only the active (dark) period was analyzed and visits lasting longer than 180 seconds were excluded from the analysis.

### Statistical analysis

Immunoassay data contained zero values and were therefore analyzed using a Mann-Whitney U test and correction was made for multiple comparisons. Cell counting data were analyzed using an unpaired Student's *t*-test. For the behavioral analysis generalized estimating equations (GEE) were used to assess the average response of the populations for the different parameters measured in the IntelliCage [46]. Data were first analyzed for a Time × Treatment effect and if no significance was achieved, time and treatment were analyzed separately. All the analyses were performed using SigmaStat 2.0 (SPSS, Chicago, IL, USA). All data are shown as mean ± standard error of the mean. *P* < 0.05 was considered statistically significant.

## Abbreviations

C3: the third complement component
CNS: central nervous system
DG: dentate gyrus
GCL: granule cell layer
IR: irradiation
P10: postnatal day 10
RT: radiotherapy
SGZ: subgranular zone
ML: molecular layer.
